# Risk factors for chronic postsurgical pain in visceral surgery: a matched case–control analysis

**DOI:** 10.1007/s00423-022-02709-z

**Published:** 2022-10-25

**Authors:** Stéphanie F. Perrodin, Win-Hua Trinh, Konrad Streitberger, Claudine Di Pietro Martinelli, Michael Alexander Harnik, Melanie Holzgang, Daniel Candinas, Guido Beldi

**Affiliations:** 1 Department of Visceral Surgery and Medicine, Inselspital, Bern University Hospital, University of Bern, Freiburgstrasse, 3010 Bern, Switzerland; 2 Department of Anaesthesiology and Pain Medicine, Inselspital, Bern University Hospital, University of Bern, Bern, Switzerland

**Keywords:** Chronic postsurgical pain, Persistent postoperative pain, Risk factors, Visceral surgery, Abdominal surgery

## Abstract

**Purpose:**

Chronic postsurgical pain (CPSP) after abdominal visceral surgery is an underestimated long-term complication with relevant impact on health-related quality of life and socioeconomic costs. Early identification of affected patients is important. We aim to identify the incidence and risk factors for CPSP in this patient population.

**Methods:**

Retrospective case–control matched analysis including all patients diagnosed with CPSP after visceral surgery in our institution between 2016 and 2019. One-to-two case–control matching was based on operation category (HPB, upper-GI, colorectal, transplantation, bariatric, hernia and others) and date of surgery. Potential risk factors for CPSP were identified using conditional multivariate logistic regression.

**Results:**

Among a cohort of 3730 patients, 176 (4.7%) were diagnosed with CPSP during the study period and matched to a sample of 352 control patients. Independent risk factors for CPSP were age under 55 years (*OR* 2.64, *CI* 1.51–4.61), preexisting chronic pain of any origin (*OR* 3.42, *CI* 1.75–6.67), previous abdominal surgery (*OR* 1.99, *CI* 1.11–3.57), acute postoperative pain (*OR* 1.29, *CI* 1.16–1.44), postoperative use of non-steroidal anti-inflammatory drugs (*OR* 3.73, *OR* 1.61–8.65), opioid use on discharge (*OR* 3.78, *CI* 2.10–6.80) and length of stay over 3 days (*OR* 2.60, *CI* 1.22–5.53). Preoperative Pregabalin intake was protective (*OR* 0.02, *CI* 0.002–0.21).

**Conclusion:**

The incidence of CPSP is high and associated with specific risk factors, some of them modifiable. Special attention should be given to sufficient treatment of preexisting chronic pain and acute postoperative pain.

**Supplementary Information:**

The online version contains supplementary material available at 10.1007/s00423-022-02709-z.

## Introduction

Chronic postsurgical pain (CPSP) is defined by the International Association for the Study of Pain (IASP) as pain in the surgical area or referred anatomical region, beginning or increasing after surgery and persisting for over 3 months after surgery, for which no other cause is identified [1, 2]. CPSP is increasingly recognized as a relevant postoperative complication, with an important impact on quality of life and relevant socio-economic implications [3, 4]. However, it is surprisingly under-investigated in patients after abdominal visceral surgery [5–8]. This stands in contrast with the need for early identification of patients at risk for CPSP in order to develop specific prevention and management strategies.

The overall incidence of CPSP in Europe varies between 2.2 and 23.6% in a heterogeneous surgical population comprising orthopaedic, gynaecological, urologic and thoracic surgery [9]. Suggested risk factors in patients after general surgery include female gender, young age, preoperative pain, preoperative psychological status and acute postoperative pain [5, 6]. Duration of surgery and reoperation for anastomotic leakage and inflammatory bowel disease were also identified as risk factors after colorectal surgery [7, 8].

The main limitations of previous studies are the following: (1) an outdated definition of CPSP, (2) not assessing its relation to pre-existing pain [5] 3) limited inclusion of variables, not addressing non-surgery-related factors [8] or not including the presence and treatment of pre- and postoperative pain [5, 7, 8].

Therefore, assessment of the incidence and risk factors for CPSP in patients undergoing abdominal surgery is needed. In particular, inclusion of patients with CPSP, according to the IASP definition with added documentation of perioperative analgesic drug use is required.

The aim of this study is thus to determine the incidence and potential risk factors for CPSP in patients after abdominal visceral surgery, according to the definition of the IASP, and matched according to the type of surgery.

## Methods

We conducted a retrospective, 1:2 matched case–control study to identify risk factors for CPSP in patients undergoing abdominal visceral surgery at the Department of Visceral Surgery and Medicine of the University Hospital Bern, Inselspital, between January 2016 and December 2019. The study protocol was approved by the Regional Ethical Review Board in Bern (KEK-Nr. 2021–01,491). General consent was obtained at time of hospitalization. Study-specific informed consent has been waived by the ethics commission due to the retrospective design.

This report adheres to the STROBE criteria for case–control studies (Suppl. Table 1) [10].

### Case selection

CPSP is defined as pain in the surgical area or referred anatomical region that develops or intensifies after surgery and lasts beyond the normal healing process, at least 3 months after surgery, for which no other cause is identified [1, 2]. The diagnostic procedures to exclude other causes of chronic pain were chosen by the doctor in charge. Cases were eligible for inclusion in the study, if CPSP was documented as a diagnosis or if the follow-up report clearly stated symptoms compatible with the definition of CPSP. In addition, there had to be a documented impact on quality of life, performance or patient management.

Excluded were patients for whom no general consent was available, who did not have an incision in the abdominal wall, where the duration of follow-up was less than 3 months or who died in the first 3 months after surgery. Patients in whom postoperative pain was described in the follow-up report as a twinge sensation or discomfort, without any impact on performance, quality of life or management, who subsequently did not need additional follow-up visits or treatment for pain were excluded. This was determined to ensure that only patients with clinically relevant CPSP diagnosis were included.

Patients who underwent repeated abdominal surgeries in less than a 3-month interval were evaluated for CPSP after the last operation.

Two of the authors evaluated all patients for inclusion independently. In case of disagreement, they reached a consensus together with a third author.

### Matching process

**Fig. 1:**
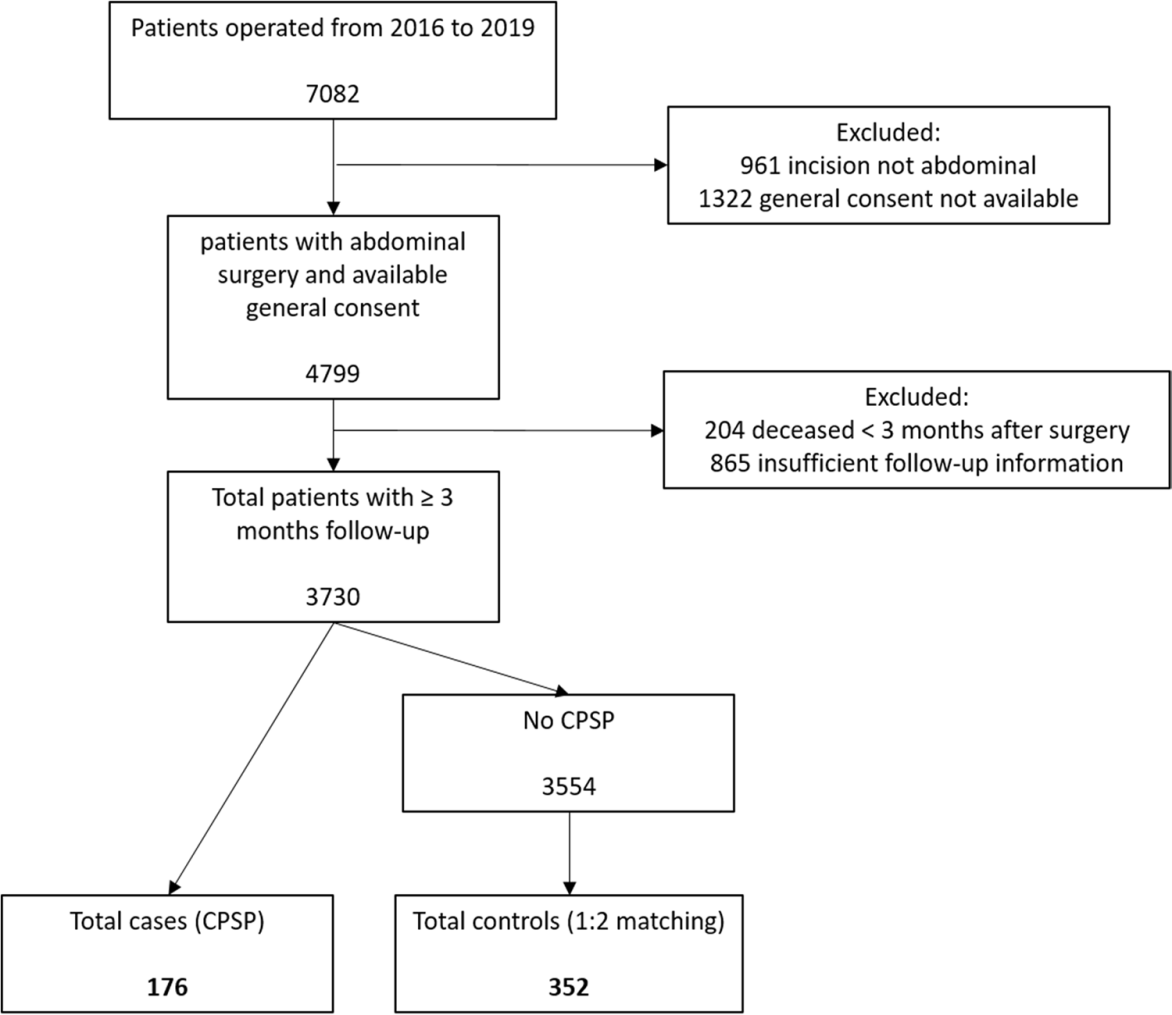
Flow-chart of patient inclusion. CPSP, chronic postsurgical pain

Control patients were randomly selected from the 3554 patients who underwent abdominal surgery, for whom follow-up of at least 3 months was available and who did not develop CPSP (Fig. 1). They were matched in a 1:2 case–control ratio according to the month of surgery (+/− 2 months from the date of the case’s surgery) and the type of surgery. They were subsequently classified according to the surgical specialty: hepato-pancreatico-biliary surgery, upper gastro-intestinal surgery, colorectal surgery, bariatric surgery, hernia repair, kidney or liver transplantation or others. Others included nephrectomies, resection of retroperitoneal tumours, splenectomies and diagnostic laparoscopies or laparotomies. They were grouped together due to the small number of cases per procedure. The surgery date was chosen as a matching factor to control for the influence of time on the changes in anaesthesia and/or surgical procedures. Matching for the type of surgery was selected to account for the different follow-up times and methods in the visceral surgery population and the various surgical techniques.

### Data collection

The following data were collected from the patient’s electronic medical records in an electronic database.

Patient’s demographics.

- Age in years
- Sex
- Body-mass index (BMI) in kg/m.^2^
- Comorbidities: metabolic disease such as diabetes and malnutrition, cardiovascular disease, inflammatory bowel disease (IBD), history of chemotherapy or radiotherapy, psychiatric comorbidities (depression, anxiety, addiction and personality disorders), previous abdominal surgery, preexisting chronic pain defined as pain present for over 3 months at the time of surgery at any location, preoperative analgesic or co-analgesic drugs

Surgery- and anaesthesia-related variables.

- Emergency or elective surgery
- Surgical technique defined as laparoscopic or open surgery and type of incision
- Mesh placement during surgery
- Duration of surgery in minutes
- Oncologic surgery
- Use of regional anaesthesia such as paravertebral block, epidural anaesthesia, local infiltration of lidocaine in the wound or transversus abdominis plane (TAP) block. Regional anaesthesia is consistently used in our institution, in addition to general anaesthesia, and TAP blocks are preferred whenever possible.
- Intraoperative analgesic drugs used

Postoperative management and outcome variables.

- Location of immediate postoperative care (recovery room, intermediate or intensive care)
- Analgesic drugs in the first 24 h. We routinely prescribe paracetamol, metamizol and opioids as needed, adding NSAIDs, ketamin and/or clonidine, if pain control is insufficient.
- Use of patient-controlled anaesthesia (PCA)
- Maximum postoperative pain on the numeric rating scale (NRS) at rest and mobilization during the first 24 h after surgery
- Postoperative complications, according to the Clavien-Dindo Classification [11]
- Length of stay in days
- Analgesic drugs at discharge

### Statistical analysis

The analysis was performed with STATA version 15.1 (Stata Corp., College Station, TX, USA). Categorical variables were compared using the Cochrane-Mantel–Haenszel test and continuous variables with a multilevel mixed-effect linear regression. This to stratify for the 1:2 matched groups. Patient-, surgery- and anaesthesia-related characteristics are presented as mean and standard deviations (SD) for continuous variables, and proportions and frequency for categorical variables. All tests were two sided. A *p*-value < 0.05 was considered statistically significant.

Univariate analysis of potential risk factors for CPSP was conducted using conditional logistic binary regression. Correction for multiple testing was performed using the Benjamini–Hochberg procedure to control the false discovery rate (FDR). All variables with a FDR-corrected *p*-value of < 0.05 were included in the initial multivariate analysis to estimate the adjusted association of said variables with CPSP. Some prespecified plausible interaction terms were added to account for possible interactions. The model was refined using a backwards stepwise selection process. The quality of the model was assessed with likelihood ratio tests and link tests. The continuous variables were tested as continuous variables, as categorical variables using restricted cubic splines and as binary variables using the significant cut-off. They were included in the model as continuous or binary variables, as appropriate according to the quality of the model. Associations are presented as odds ratios (*OR*) and adjusted odds ratios (*aOR*) with their 95% confidence intervals (*CI*).

## Results

**Fig. 2:**
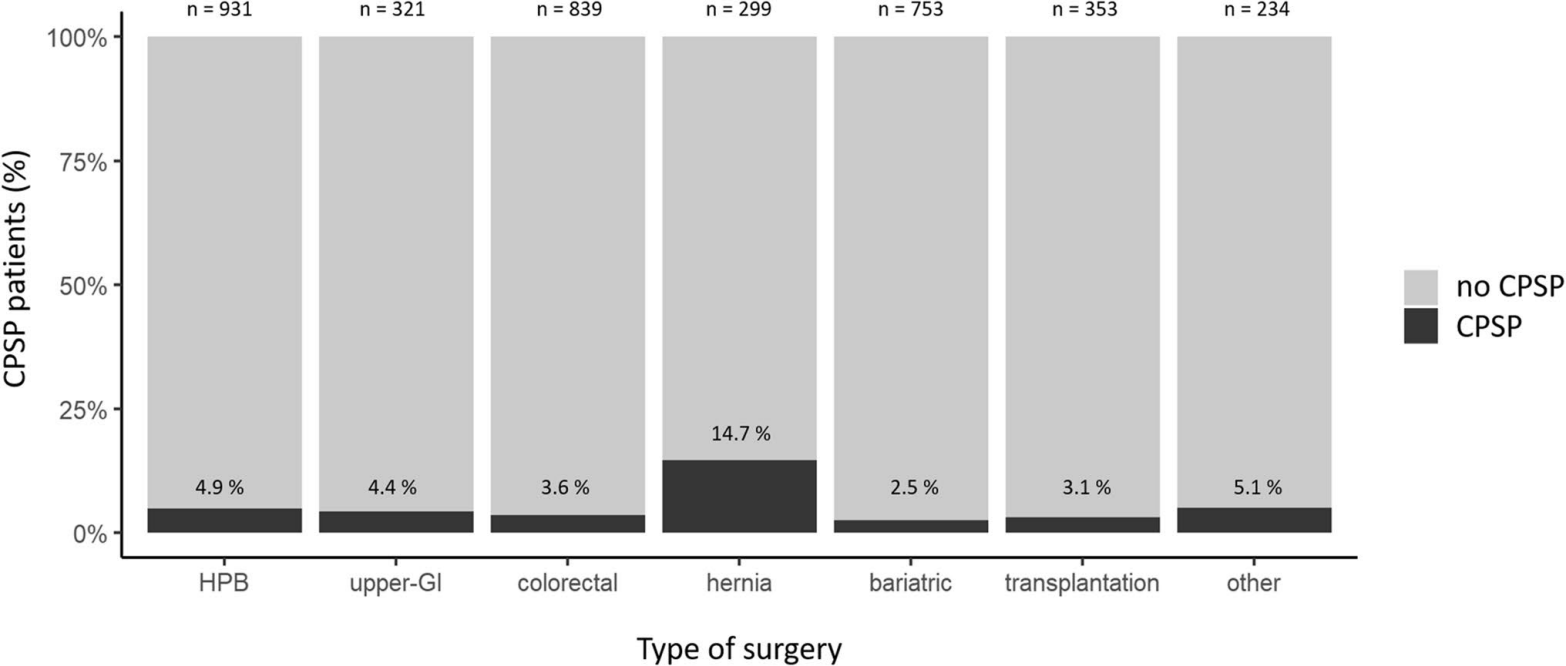
Proportion of CPSP patients per type of surgery. CPSP, chronic postsurgical pain

Of the 7082 patients who underwent surgery in our institution between 2016 and 2019, 3730 met the inclusion criteria, and 176 CPSP cases were identified. The flow chart of patient selection is shown in Fig. 1. The incidence of CPSP between 2016 and 2019 was 4.7% (176/3730) among the patients for whom follow-up information of at least 3 months was available. We observed comparable yearly incidences, with 4.9% (50/1015) in 2016, 5.3% (33/621) in 2017, 3.3% (28/856) in 2018 and 5.3% (65/1238) in 2019. The proportion of CPSP patients per type of surgery is shown in Fig. 2.

Patient characteristics are reported in Table 1; characteristics of surgery and anaesthesia in Table 2. The proportion of female patients was higher in patients with CPSP (55.1% versus 41.5%, *p* = 0.002), and they were significantly younger in age (mean 51.4 versus 58.6 years, *p* = 0.000). There was no difference in psychiatric comorbidities, except in addiction and personality disorders. The number of patients with previous abdominal surgery was high in both groups and significantly higher in the CPSP group (69.9% versus 58%, *p* = 0.004). All surgical variables were equally distributed in both groups, as they were dictated by the type of surgery, which was a matching criterion. All patients underwent general anaesthesia, and additional regional anaesthesia was used equally frequently in both groups (68.7% and 68.2% in the CPSP and non-CPSP groups, respectively, *p* = 0.866). Table 3 reports the postoperative management and surgical outcome parameters. Although there were more complications overall in the CPSP group (41.5% versus 33%, *p* = 0.035), the proportion of severe complications (Dindo-Clavien ≥ IIIB) was similar in both groups (30.1% versus 37.1%, *p* = 0.288). Mean maximal pain at rest was significantly higher in the CPSP group than the control group (5.8 (SD 2.4) versus 3.8 (SD 2.5), *p* =  < 0.001), as was mean maximal pain at mobilization (7.1 (SD 2.02) versus 5.3 (SD 2.2), *p* =  < 0.001).

**Table 1:** Patient characteristics (*n* = 528)

	CPSP (*n* = 176)	No CPSP (*n* = 352)	*p*-values*
Female sex, *n* (%)	97 (55.1)	146 (41.5)	0.002
Age in years, mean (SD)	51.4 (14.5)	58.6 (16.4)	< 0.001
Age < 55 years, *n* (%)	96 (54.6)	122 (34.7)	< 0.001
BMI, mean kg/m^2^ (SD)	27 (6.7)	27.4 (6.7)	0.349
BMI < 35 kg/m^2^, *n* (%)	155 (90.1)	294 (85.7)	0.043
Comorbidities, *n* (%)	106 (60.2)	219 (62.2)	0.654
None	70 (39.8)	133 (37.8)	0.654
Metabolic disease (diabetes, malnutrition)	22 (12.5)	50 (14.2)	0.596
Cardiovascular disease	65 (36.9)	155 (44)	0.118
Rheumatoid disease (arthritis, etc.)	13 (7.4)	25 (7.1)	0.905
History of chemotherapy and/or radiotherapy	18 (10.2)	54 (15.3)	0.092
Inflammatory bowel disease	7 (4)	13 (3.7)	0.871
Psychiatric comorbidities, *n* (%)	43 (24.4)	63 (17.9)	0.074
Depression	29 (16.5)	47 (13.4)	0.327
Anxiety	4 (2.3)	13 (3.7)	0.361
Other (addiction, personality disorders)	20 (11.4)	21 (6)	0.027
Previous abdominal surgery, *n* (%)	123 (69.9)	204 (57.95)	0.004
Number of previous abdominal surgeries, mean (SD)	1.6 (2)	1.2 (1.5)	0.001
Previous laparotomy, *n* (%)	72 (40.9)	123 (34.9)	0.143
Previous laparoscopy, *n* (%)	85 (48.3)	117 (33.2)	0.001
Previous mesh implantation, *n* (%)	18 (10.2)	37 (10.5)	0.919
Preoperative chronic pain, *n* (%)	65 (36.9)	50 (14.2)	< 0.001
Abdominal pain	32 (18.2)	18 (5.1)	< 0.001
Inguinal pain	4 (2.3)	2 (0.6)	0.058
Musculoskeletal pain	34 (19.3)	34 (9.7)	0.001
Duration in months, mean (SD)	59.95 (74.8)	101.02 (109.5)	0.025
Preoperative analgesic intake, *n* (%)	62 (35.2)	32 (9.1)	< 0.001
Opioids	22 (12.5)	13 (3.7)	< 0.001
Preoperative co-analgesic intake, *n* (%)	48 (27.3)	70 (19.9)	0.058
Antidepressants	34 (19.3)	49 (13.9)	0.121
Antiepileptics	7 (4)	7 (2)	0.186
Myorelaxants	0	2 (0.6)	0.317
Pregabaline	1 (0.6)	18 (5.1)	0.009
Gabapentine	3 (1.7)	1 (0.3)	0.077
Steroids	2 (1.1)	14 (4)	0.077

*SD* standard deviation, *BMI* body-mass index

^*^Cochrane-Mantel–Haenszel test for categorical variables, multilevel mixed-effect linear regression for continuous variables

**Table 2:** Characteristics of surgery and anaesthesia (*n* = 528)

	CPSP (*n* = 176)	No CPSP (*n* = 352)	*p*-values*
Type of surgery, *n* (%)			1.000
HPB	46 (26.1)	92 (26.1)	
Upper-GI	14 (8)	28 (8)	
Colorectal	30 (17.1)	60 (17.1)	
Hernia	44 (25)	88 (25)	
Inguinal hernia	8 (4.6)	16 (4.6)	
Transplantation	11 (6.3)	22 (6.3)	
Bariatric	19 (10.1)	38 (10.1)	
Other	12 (6.8)	24 (6.8	
Emergency surgery (versus elective), *n* (%)	31 (17.6)	68 (19.3)	0.460
Open surgery (versus laparoscopic), *n* (%)	98 (55.7)	185 (52.6)	0.149
Midline laparotomy	45/98 (45.9)	79/185 (42.7)	0.518
Subcostal laparotomy	41/98 (41.8)	82/185 (44.3)	0.424
Other open access (inguinal, pararectal)	12/98 (12.2)	24/185 (12.97)	0.876
Use of a mesh, *n* (%)	47 (26.7)	90 (25.6)	0.157
Oncologic surgery, n (%)	40 (22.7)	100 (28.4)	0.039
Duration of surgery in minutes, mean (SD)	153.9 (111.8)	152.9 (98.1)	0.931
Additional use of regional anaesthesia, *n* (%)			
None	55 (31.3)	112 (31.8)	0.866
Paravertebral block	1 (0.6)	1 (0.3)	0.617
Epidural anaesthesia	47 (26.7)	93 (26.4)	0.924
Local infiltration of lidocaine in the wound	3 (1.7)	10 (2.8)	0.371
TAP block	70 (38.8)	136 (38.6)	0.728
Intraoperative analgesic drugs, *n* (%)			
Metamizol	113 (64.2)	247 (70.2)	0.134
NSAID	3 (1.7)	2 (0.57)	0.206
Opioid	173 (98.3)	350 (99.4)	0.206
Clonidine	27 (15.3)	31 (8.8)	0.021
Ketamine	45 (25.6)	104 (29.6)	0.286
Dexmedetomidine	20 (11.4)	64 (19.2)	0.031

*SD* standard deviation, *HPB* hepato-biliary-pancreatic surgery, *upper-GI* upper gastro-intestinal surgery, *TAP block* transversus abdominis plane block, *NSAID* non-steroidal anti-inflammatory drugs

^*^Cochrane-Mantel–Haenszel test for binomial variables, multilevel mixed-effect linear regression for continuous variables

**Table 3:** Postoperative management and outcomes (*n* = 528)

	CPSP (*n* = 176)	No CPSP (*n* = 352)	*p*-values*
Immediate postoperative care, *n* (%)			
Recovery room	108 (61.4)	205 (58.2)	0.294
Intermediate care unit	55 (31.3)	118 (33.5)	0.446
Intensive care unit	14 (7.95)	31 (8.8)	0.627
Postoperative analgesic drugs in the first 24 h, *n* (%)			
Paracetamol	135 (77.1)	286 (81.3)	0.194
Metamizol	139 (79.4)	300 (85.2)	0.091
Opioids	131(74.9)	243 (69)	0.138
NSAID	54 (31)	59 (16.8)	< 0.001
Clonidine	11 (6.3)	6 (1.7)	0.006
Ketamin	7 (4)	7 (2)	0.186
Pregabalin	1 (0.6)	10 (2.8)	0.088
Gabapentine	0	3 (0.9)	0.221
Dexmedetomidine	2 (1.1)	0	0.046
Patient controlled analgesia, *n* (%)	37 (21.1)	53 (15.1)	0.076
Epidural anesthesia, n (%)	48 (27.4)	96 (27.3)	1.000
Maximum pain on the NRS during the first 24 h, mean (SD)			
At rest	5.8 (2.4)	3.8 (2.5)	< 0.001
During mobilization	7.1 (2.02)	5.3 (2.2)	< 0.001
Postoperative complication, *n* (%)	73 (41.5)	116 (33)	0.035
Dindo-Clavien ≥ IIIB	22 (30.1)	43 (37.1)	0.288
Length of stay in days, mean (SD)	10.4 (13.4)	8.2 (8.3)	0.006
On the ward	8.9 (11.3)	6.8 (6.4)	0.001
In intensive or intermediate care	1.4 (3.8)	1.4 (3.5)	0.080
Pain treatment at discharge, *n* (%)			
None	5 (2.8)	13 (3.7)	0.617
Paracetamol	142 (80.7)	268 (76.1)	0.212
Metamizol	129 (73.3)	244 (69.3)	0.360
NSAID	19 (10.8)	35 (9.9)	0.740
Opioids	97 (55.1)	126 (35.8)	< 0.001

*SD* standard deviation, *NSAID* non-steroidal anti-inflammatory drugs, *NRS* numeric rating scale

^*^Cochrane-Mantel–Haenszel test for categorical variables, multilevel mixed-effect linear regression for continuous variables

**Fig. 3:**
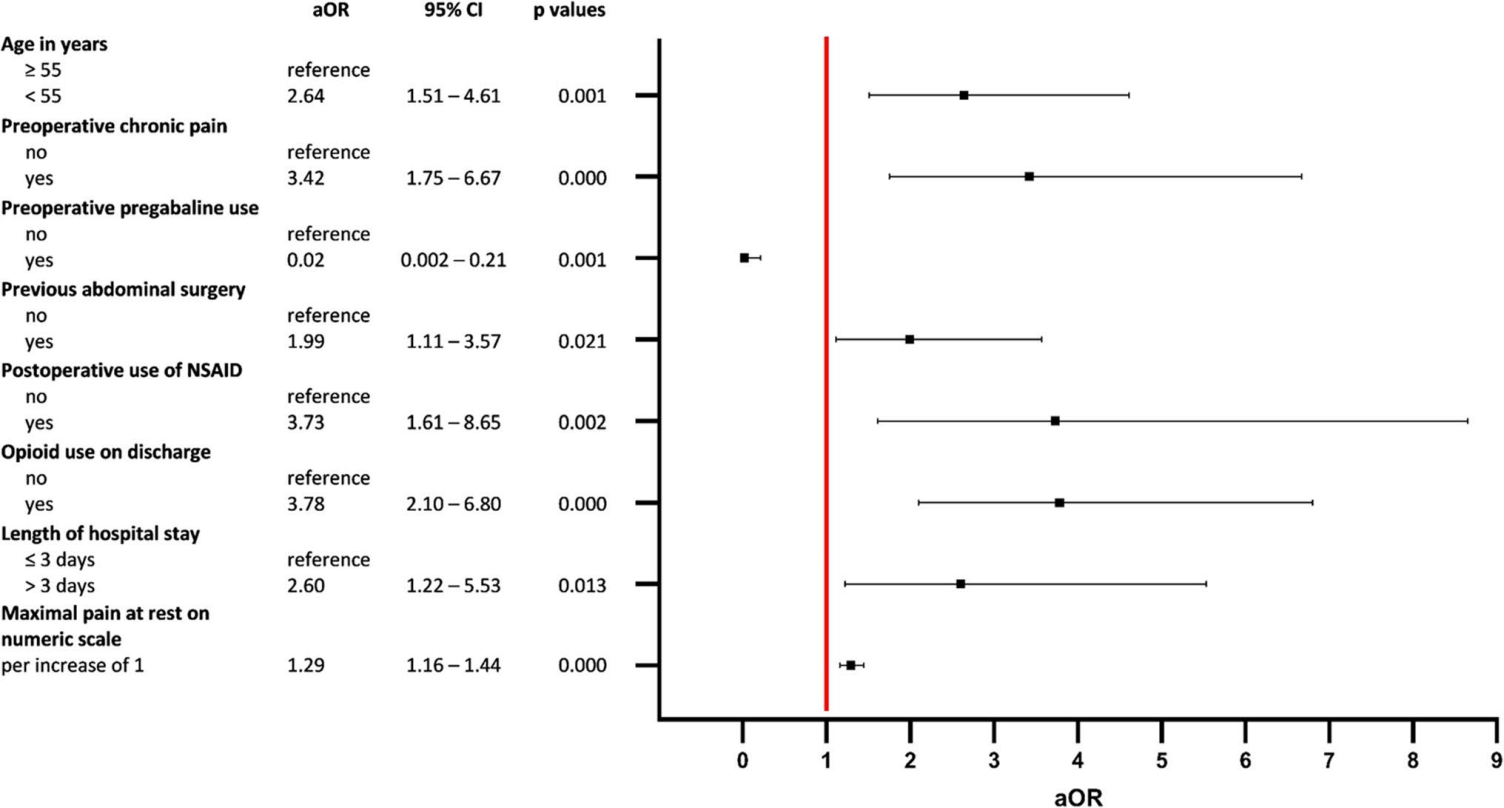
Results of the multivariate analysis indicating factors associated with CPSP. Vertical line: null effect (*aOR* = 1). *aOR*, adjusted odds ratios; *CI*, confidence interval; *NSAID*, non-steroidal anti-inflammatory drugs; *CPSP*, chronic postsurgical pain

From the univariate analysis (Table 4), the independent risk factors for CPSP are identified in the multivariate analysis in Fig. 3. Age under 55 years, pre-existing chronic pain, previous abdominal surgery, the use of NSAID in the early postoperative phase, the use of opioids on discharge, higher maximal pain at rest on the numeric scale and a length of stay over 3 days were independently associated with CPSP. Preoperative Pregabalin use, on the other hand, seemed to be associated with less CPSP.

**Table 4:** Univariate analysis

	Odds ratio	95% *CI*	*p*-values*	pFDR^±^
Sex female vs male	1.85	1.25–2.74	0.002	0.005
Age < 55 vs ≥ 55	2.57	1.71–3.87	< 0.001	< 0.001
BMI < 35 vs ≥ 35	2.22	1.01–4.89	0.047	0.061
Psychiatric comorbidities	1.50	0.96–2.34	0.076	0.091
Addiction and personality disorders	2.07	1.07–3.99	0.030	0.047
Previous abdominal surgery	1.7996	1.196–2.71	0.005	0.010
Number of abdominal surgeries (per additional surgery)	1.2	1.07–1.37	0.002	0.005
Previous laparoscopy	1.95	1.33–2.86	0.001	0.003
History of chronic pain	3.95	2.46–6.35	< 0.001	< 0.001
Duration of pre-existing chronic pain (per additional month)				
None	Reference			
≤ 12 months	9.34	3.77–23.13	< 0.001	< 0.001
> 12 months	2.36	1.31–4.23	0.004	0.009
Preoperative analgesic intake	5.26	3.19–8.67	< 0.001	< 0.001
Preoperative opioid intake	3.56	1.76–7.21	< 0.001	< 0.001
Preoperative co-analgesic intake	1.49	0.98–2.26	0.059	0.074
Preoperative pregabalin intake	0.11	0.015–0.83	0.032	0.047
Preoperative gabapentin intake	6	0.62–57.68	0.121	0.125
Preoperative steroids intake	0.29	0.06–1.26	0.097	0.104
Oncological resection	0.52	0.27–0.98	0.004	0.009
Intraoperative catapressan administration	1.92	1.09–3.37	0.024	0.04
Intraoperative dexmedetomidine administration	0.53	0.298–9.95	0.033	0.047
Postoperative metamizol intake	0.66	0.41–1.07	0.093	0.103
Postoperative NSAID intake	3.43	1.95–6.05	< 0.001	< 0.001
Postoperative catapressan intake	3.67	.36–9.91	0.010	0.018
Postoperative pregabalin intake	0.2	0.03–1.56	0.125	0.125
PCA	1.54	0.95–2.50	0.079	0.091
Opioids on discharge	4.73	2.92–7.65	< 0.001	< 0.001
LOS > 3 versus ≤ 3	1.99	1.22–3.25	0.006	0.011
Maximal pain at rest on NRS				
For each increase of 1	1.41	1.29–1.54	< 0.001	< 0.001
≥ 4 versus < 4	5.17	3.17–8.38	< 0.001	< 0.001
Maximal pain during mobilization on NRS				
For each increase of 1	1.50	1.35–1.66	< 0.001	< 0.001
≥ 4 versus < 4	5.32	2.59–10.90	< 0.001	< 0.001
Postoperative complication	1.56	1.03–2.36	0.036	0.049

*CI* confidence interval, *pFDR* false discovery rate *p*-value, *BMI* body mass index, *NSAID* non-steroidal anti-inflammatory drugs, *PCA* patient controlled analgesia, *LOS* length of stay, *NRS* numeric rating scale

^*^Univariate analysis using logistic binary regression

^±^Benjamini–Hochberg procedure-adjusted *p*-value to control for false discovery rate

## Discussion

The four-year incidence of CPSP was 4.7% in this study, which is lower than the 17 to 32% reported in the studies on colorectal surgery [7, 8, 12]. These differences could be explained by the study designs, the time of data collection and the definition of CPSP [5, 7, 8]. The proportion of patients with CPSP varies between 2.5 and 5.1% for most types of surgeries in this series, except for patients after hernia repair, where it is higher (14.7%). Chronic neuropathic pain is frequent after hernia repair [13]. In our clinic, patients who underwent hernia surgery are not routinely seen for a follow-up visit, and data is often lacking at the 3 months cut-off used for inclusion in this study. The increased proportion of CPSP in this subgroup could thus be partially caused by the lack of systematic follow-up in patients without CPSP.

We found that age under 55 years, preexisting chronic pain, previous abdominal surgery, the use of NSAID in the immediate postoperative phase, the use of opioids on discharge, a length of stay over 3 days and a higher intensity of postoperative pain are independent risk factors for CPSP.

The risk of CPSP is increased in younger patients, as shown consistently before [6, 7, 14]. While female sex was not an independent risk factor in the multivariate analysis, there were significantly more female patients in the CPSP group. Evidence for female or male sex as a risk factor is inconsistent [14]. One possible explanation is mediation of the effect of sex through the effect of the intensity of acute postoperative pain, as shown by Mi et al. [15], but in our study those two factors were independent. There was also no correlation between sex and postoperative analgesic drug administration.

Previous abdominal surgery was predicting CPSP. While studies on patients after colorectal surgery specifically found no difference [7, 8], studies on other abdominal surgeries [6, 16–18] found an association of previous surgery with CPSP. Whether lower individual pain thresholds influenced the indication for previous surgeries and thus the number of surgeries a patient undergoes cannot be evaluated with this analysis. But having had previous surgery was not associated with the level of acute postoperative pain. This suggests that it might be unrelated to the individual pain threshold.

Our study identifies preexisting chronic pain, both at the site of surgery and elsewhere, as a risk factor for CPSP — this corroborates previous research [16, 19]. Nerve damage during surgery with acute postoperative pain resulting in central sensitization and neuropathic pain syndromes is one of the suspected mechanism of CPSP [6, 18, 20]. A possible explanation for preexisting chronic pain, in addition to previous abdominal surgery as risk factors for CPSP, is that the changes in the central nervous system caused by sensitization precede surgery. This could influence the development of CPSP after the new injury, as could patient-specific decreased pain inhibition mechanisms or preoperative use of analgesics [20, 21].

Consistently with previous research, a higher intensity of acute postoperative pain in the first 24 h after surgery was identified as a risk factor for CPSP. Liu et al. showed that factors associated with acute postoperative pain intensity were anxiety and higher consumption of analgesics [22]. In our study, patients with higher acute pain intensity also had higher in-hospital use of opioids, but psychological factors did not play a significant role. If CPSP is a result of sensitization, then stricter control of acute postoperative pain through adapted analgesic dosage should participate in its prevention.

Multiple interventions to treat acute postoperative pain have been investigated, with various results. In our institution, we consistently use regional anaesthesia techniques, in addition to general anaesthesia. TAP blocks are preferred whenever possible. While they are efficient to reduce acute postoperative pain in the initial phase, they have no effect on preventing CPSP [12, 23, 24]. There was no significant difference between the groups in perioperative pain management, except for the use of NSAID. NSAID use was associated with CPSP, independently from the level of postoperative pain on the NRS. NSAID are not routinely used in our department in the immediate postoperative phase, as described in the “Methods.” We found no data on postoperative NSAID use and CPSP in the literature, but the use of at least two combined non opioid analgesic drug classes during surgery does reduce acute postoperative pain [25]. In our cohort, the level of reported acute postoperative pain was similar in patients who received NSAID in the first 24 h after surgery and those who did not. NSAID could be a surrogate factor for initial refractory postoperative pain, where additional analgesic drugs did control the postoperative situation, but with no beneficial impact on the risk of CPSP at a later time-point. A recent study suggested that the resolution of acute pain is mediated by an active immune process rather than a progression of acute pain to chronic pain [26]. In patients with chronic pain, this neutrophile-activated inflammatory response would be impaired. Using NSAID or steroids for acute pain, while beneficial in the short-term, would prevent the initiation of an appropriate inflammatory response and thus lead to chronic pain [26]. The use of ketamine has been linked to prevention of CPSP by blocking N-methyl-D-aspartate-receptors and preventing central sensitization. Its intraoperative use in colorectal surgery led to a lower incidence of CPSP in this population [8, 27], but the effect is not consistent [28]. We did not observe it in our patients, whether for intra- or postoperative use. Dexmedetomidine, a highly selective α2 adrenoreceptor agonist, reduces postoperative pain and opioid use in the acute phase [29]. In a prospective study, it was also associated with significantly less CPSP [30]. In our study, the use of dexmedetomidine peri- and postoperatively was significantly lower in the CPSP group but is not significant in the multivariate analysis. This is probably due to the overall low number of patients for which it was used. We do not administer it systematically but tend to do so in patients with a history of chronic pain or substance abuse.

Patients under Pregabalin at time of surgery seemed to have a lower risk of developing CPSP in this study, for which we did not find a correlation in the literature [28]. A possible explanation for this observed effect in our cohort is that in these patients, preexisting chronic pain might be better controlled in the preoperative phase. Better preoperative pain control has been linked to lower risk of CPSP in orthopaedic surgery [31]. It might also be that these patients have more frequent follow-up visits in the postoperative phase, be it from a pain specialist, their surgeon or their general practitioner. This would mean that they are more likely to be included in this study, as having follow-up information 3 months after surgery was an inclusion criterion. The relatively high proportion of patients under Pregabalin in the control group speaks for the quality of the selection, where the control group is not just constituted of otherwise healthy patients.

We found no correlation between depression or anxiety and CPSP. In the literature, there is evidence for psychological factors such as depression, anxiety and catastrophising as predictors of CPSP [4, 7, 18, 21]. Althaus et al., on the other hand, found no correlation between anxiety and CPSP in a mixed population of patients who underwent orthopaedic surgery, general surgery, visceral surgery and neurosurgery [32]. VanDenKerkhof et al. conducted a prospective study on women who underwent gynaecological surgery, where depression and anxiety were not correlated to CPSP [33]. The importance of these factors could depend on the studied population. More likely, because our patients did not undergo a detailed psychological assessment, the presence or absence of psychological comorbidities was taken from the list of diagnosis, which is less precise.

Our study is limited by its retrospective nature. Because we do not systematically see all patients in the outpatient clinic after surgery, 18% of all patients who underwent abdominal surgery during this timeframe never had any kind of follow-up visit and had to be excluded. It is also possible that lower intensity pain might not be reported by the patient and/or the physician, and some patients were falsely classified as having no CPSP.

In conclusion, the identification of these risk factors allows for a preventive and personalized health care approach to our at-risk patients. We emphasized the importance of the optimization of pre- and postoperative management, with special attention to sufficient treatment of preexisting chronic pain and acute postoperative pain with a multimodal analgesic approach. Finally, an additional follow-up consultation 3 months after surgery should be considered in at-risk patients to avoid a delay in diagnosing CPSP and uncontrolled and inadequate long-term opioid use.

## Supplementary Information

Below is the link to the electronic supplementary material.

Supplementary file1 (DOCX 31 KB)
